# Acute Laryngeal Dystonia Following Haloperidol Administration: Early Recognition Prevents Intubation

**DOI:** 10.7759/cureus.105248

**Published:** 2026-03-15

**Authors:** Saman Najib, Mehwish Zehra

**Affiliations:** 1 Internal Medicine, Nawaz Sharif Medical College, Gujrat, PAK; 2 Internal Medicine, Jinnah Sindh Medical University, Karachi, PAK

**Keywords:** acute laryngeal dystonia, anticholinergics, extrapyramidal symptoms, haloperidol, neuroleptic side effects, respiratory distress

## Abstract

Acute laryngeal dystonia (ALD) is a rare but potentially fatal extrapyramidal adverse effect of high-dose antipsychotic medications like haloperidol. It presents with sudden contraction of laryngeal muscles that causes narrowing of the airway, which rapidly progresses to respiratory failure if not promptly recognized. We report a 45-year-old male patient with schizophrenia who developed respiratory distress and inspiratory stridor within 15 minutes of receiving haloperidol for postoperative agitation following emergent abdominal surgery. Initial management with epinephrine failed to improve symptoms. Given the temporal association with haloperidol administration, an acute dystonic reaction was suspected, and the patient was promptly treated with intravenous diphenhydramine and benztropine, resulting in immediate improvement of respiratory distress. Anticholinergic therapy was continued for one week with no recurrence of symptoms. This case highlights the importance of early recognition of ALD in postoperative and critical care settings, as timely administration of anticholinergic agents can be life-saving and may prevent unnecessary endotracheal intubation. It is recommended to continue anticholinergic agents for a prolonged period of time, even after haloperidol discontinuation, to prevent the recurrence of symptoms that have been reported in many cases in the past.

## Introduction

Acute dystonia is an often painful medication-induced movement disorder involving involuntary, sustained muscle contractions, most caused by dopamine receptor antagonists like antipsychotics (e.g., haloperidol) and antiemetics (e.g., metoclopramide). Reported symptoms include torticollis (neck twist), oculogyric crisis (eyes upward), trismus (jaw lock), and laryngeal dystonia, usually occurring within 96 hours (about four days) of drug initiation or dose increase. The proposed mechanism is that antipsychotics cause dopamine receptor blockade that increases cholinergic stimulation in the basal ganglia. Other agents, including anti-malarial, antidepressants, antihistamines, and anticonvulsants, have also been implicated in cases of acute dystonic reaction [1]. Acute dystonic reactions due to antipsychotic medication occur at rates of 3-10%. An analysis of pooled data from reported cases of acute dystonic reactions suggests rates as high as 51.2% for high-potency first-generation neuroleptic agents [2]. Risk factors for acute dystonic reaction are male gender, young age, previous episode of acute dystonia, or recent cocaine use. Additional risk factors reported in the literature include alcoholism, hyperthyroidism, hypoparathyroidism, hypocalcemia, dehydration, mononucleosis, AIDS, uremia, acute respiratory infection, and stress [3]. Laryngeal dystonia is due to the involuntary contraction of the vocal cord muscle. The diagnosis of acute laryngeal dystonia (ALD) is clinical, i.e., based on characteristic symptoms (dyspnea, dysphonia, and stridor) during antipsychotic medication initiation or dose increment [4]. Our case report illustrates the development of ALD in a 45-year-old male patient after haloperidol administration that was promptly diagnosed and treated with diphenhydramine and benztropine. Early recognition and treatment prevented the need for endotracheal intubation and other invasive airway procedures. 

## Case presentation

A 45-year-old male patient with a history of schizophrenia presented to the emergency department with acute-onset abdominal pain, nausea, and multiple episodes of vomiting. The abdominal pain was diffuse and progressively worsening. A contrast-enhanced computed tomography (CT) scan of the abdomen demonstrated findings consistent with small bowel obstruction complicated by bowel ischemia. Given the severity of the imaging findings and clinical deterioration, the patient was taken emergently to the operating room. An exploratory laparotomy was performed under general anesthesia, and the patient was intubated intraoperatively. The Hartmann’s procedure was carried out due to ischemic bowel involvement. He was extubated at the end of the procedure without any immediate complications. Postoperatively, the patient was transferred to the intensive care unit (ICU) for further management and close monitoring. 

The patient had been maintained on oral olanzapine 10 mg once daily for schizophrenia prior to admission. However, this medication was held postoperatively due to nil per oral (NPO) status following surgery. His last documented dose was administered approximately 24 hours prior to surgery. On post-operative day one, the patient developed significant psychomotor agitation, characterized by restlessness, irritability, and difficulty cooperating with medical care. At that time, vital signs revealed heart rate 110 beats/min (reference values: 60-100 beats/min), blood pressure 130/90 mmHg (reference values: 90/60-120/80 mmHg), respiratory rate 13 breaths/min (reference values: 12-20 breaths/min), and temperature of 98 °F (reference values: 97.7-99.5 °F). Laboratory evaluation showed complete blood count, metabolic profile, and thyroid tests within normal limits (Table 1).

**Table 1:** Investigations done at presentation

Investigation	Patient value	Reference range
Complete blood count		
Hemoglobin	13.6 g/dL	12–16 g/dL
White blood cell count	7.2 ×10^9^/L	4.0–11.0 ×10^9^/L
Platelet count	260 ×10^9^/L	150–450 ×10^9^/L
Mean corpuscular volume (MCV)	88 fl	80–100 fl
Basic metabolic panel		
Sodium	139 mmol/L	135–145 mmol/L
Potassium	4.2 mmol/L	3.5–5.0 mmol/L
Chloride	102 mmol/L	96–106 mmol/L
Bicarbonate	24 mmol/L	22–28 mmol/L
Blood urea nitrogen	14 mg/dL	7–20 mg/dL
Creatinine	0.9 mg/dL	0.6–1.3 mg/dL
Glucose	95 mg/dL	70–100 mg/dL
Thyroid function tests		
Thyroid stimulating hormone (TSH)	2.1 μIU/mL	0.4–4.0 μIU/mL
Free T4	1.2 ng/dL	0.8–1.8 ng/dL
Free T3	3.2 pg/mL	2.3–4.2 pg/mL

Given his underlying psychiatric history and acute agitation in setting of antipsychotic interruption, intravenous haloperidol 2 mg was administered. Approximately 15 minutes following the administration of haloperidol, the patient developed sudden onset respiratory distress, characterized by inspiratory stridor, labored breathing, and visible use of accessory muscles. Oxygen saturation began to decline, and the patient appeared anxious. Given the acute presentation of stridor and recent extubation, post-extubation laryngeal edema was initially considered as a possible cause, prompting treatment with racemic epinephrine but there was no significant improvement in symptoms. The absence of response to epinephrine combined with recent administration of a high-potency dopamine antagonist, raised suspicion of acute dystonic reaction involving the laryngeal musculature. 

**Figure 1:**
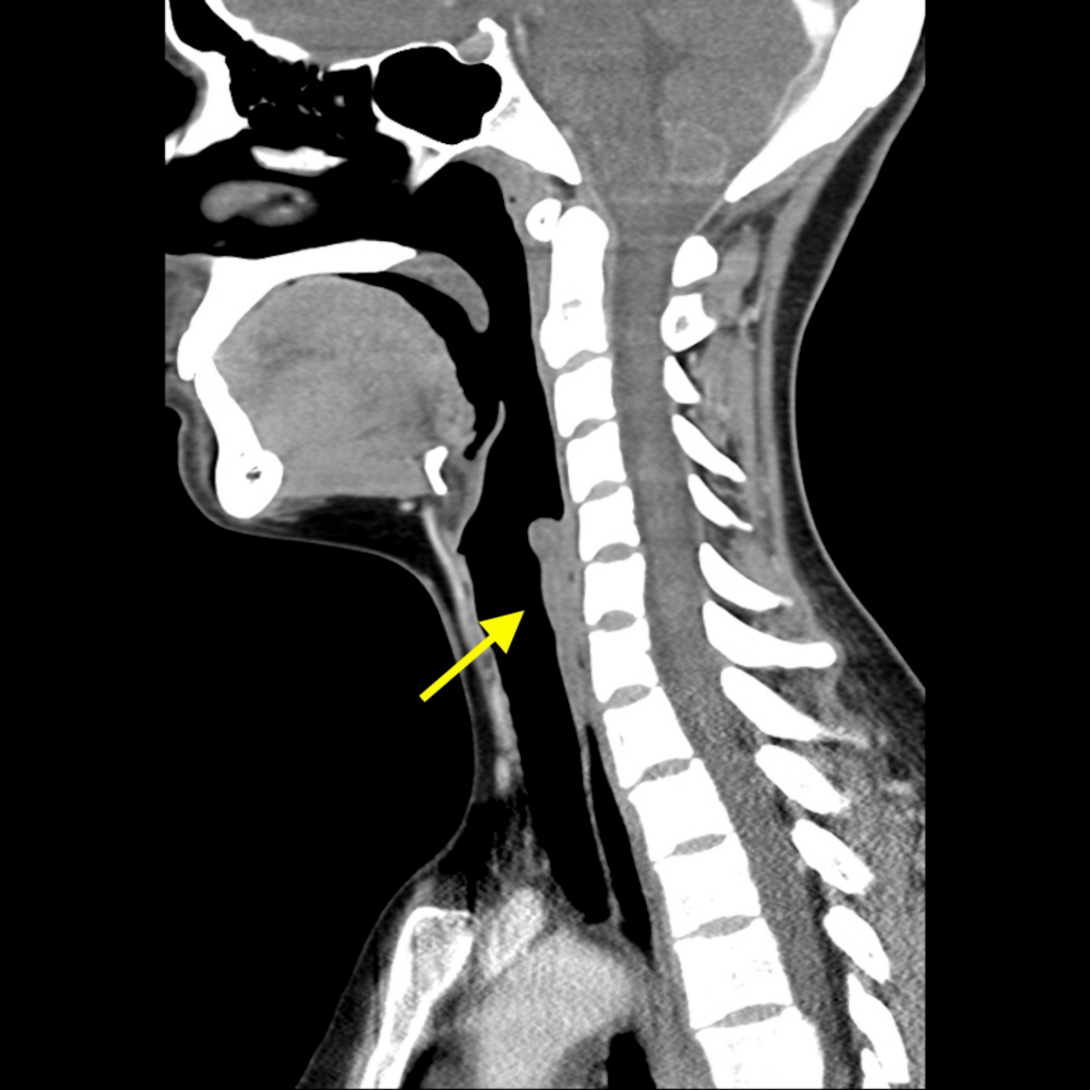
CT scan neck, sagittal section Arrow demonstrating a patent airway, normal epiglottis and symmetric prevertebral soft tissues. Cervical vertebrae are aligned normally with no evidence of mass, edema or lymphadenopathy.

**Figure 2:**
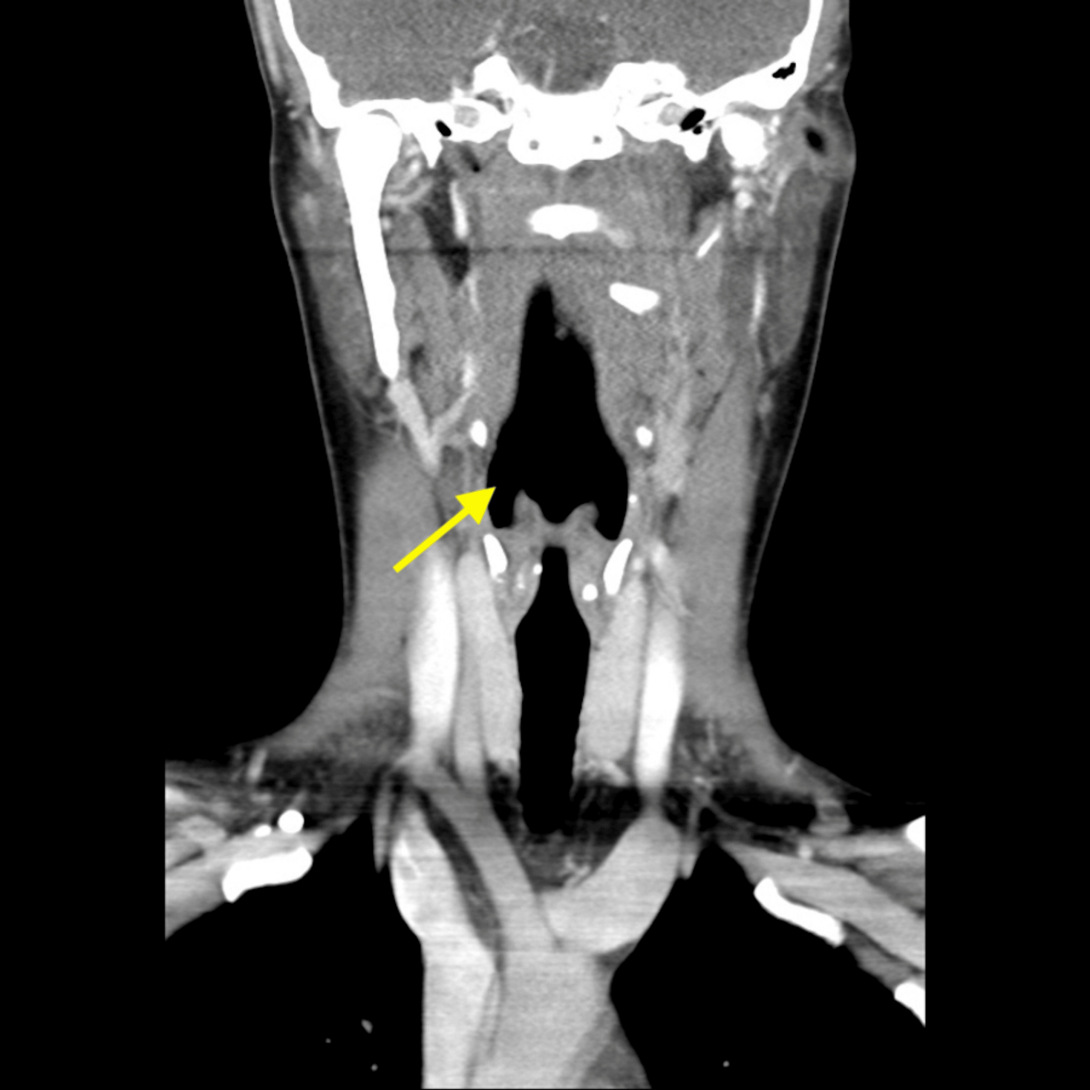
CT scan of neck, coronal view Showing a clear tracheal air column with normal epiglottis and no prevertebral soft tissue swelling. Arrow demonstrating a patent airway.

**Figure 3:**
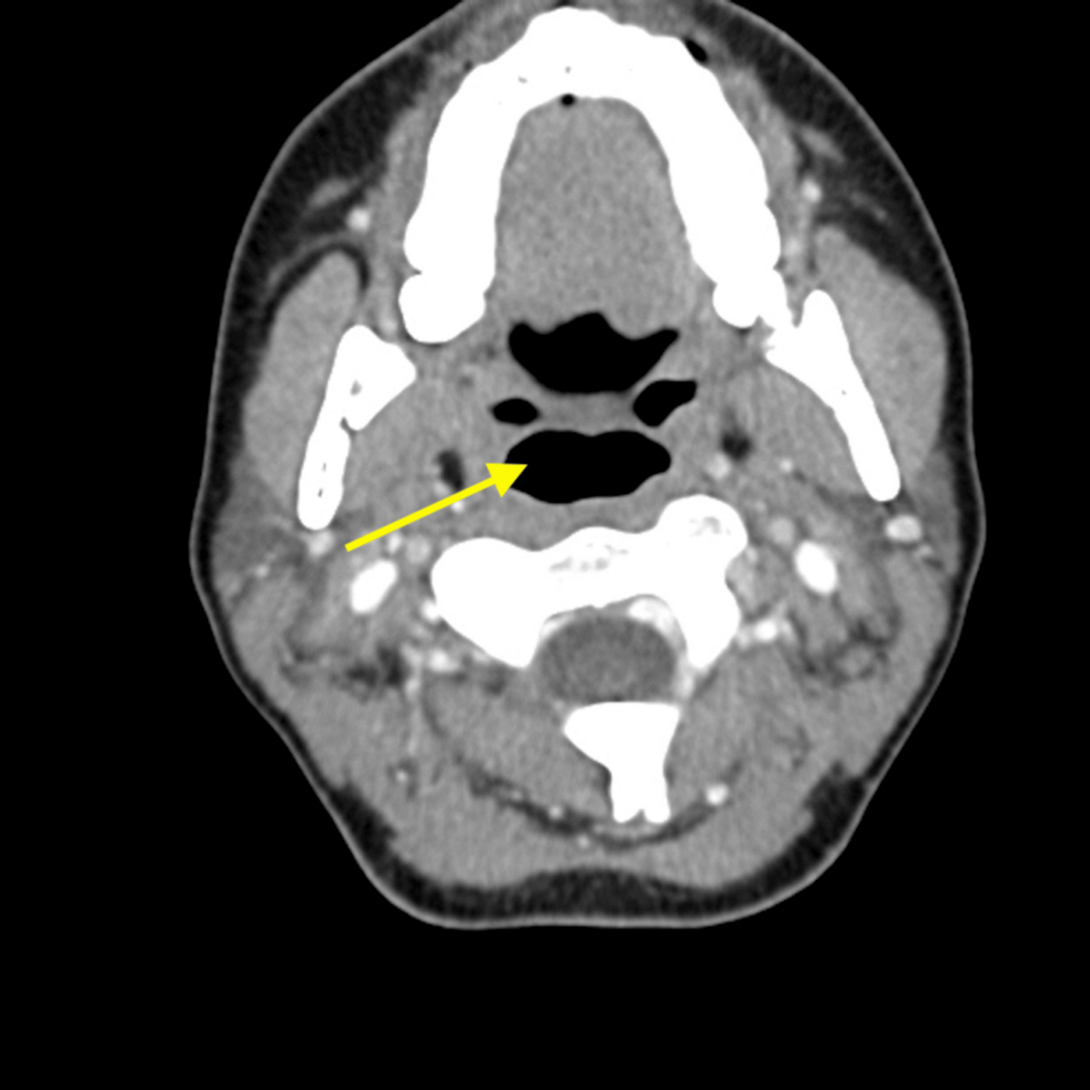
CT scan of neck, axial view Arrow demonstrating patent airway without evidence of obstruction.

The patient was then subsequently treated with diphenhydramine 50 mg IV and benztropine 1 mg IV. Within minutes of administration, there was rapid and complete resolution of stridor and respiratory distress, strongly supporting the diagnosis of haloperidol-induced ALD. However, given the patient's recent endotracheal intubation during exploratory laprotomy, post-extubation airway edema or structural airway injury remained a consideration. Therefore, a contrast enhanced CT scan of the neck was performed to exclude structural causes of airway obstruction, which demonstrated normal findings (Figures 1-3). 

The patient was continued on anticholinergics for a week till discharge, with no recurrence of symptoms. He was discharged in a stable condition.

## Discussion

Acute dystonia is an extrapyramidal adverse effect primarily mediated through dopamine D2 receptor antagonism in the nigrostriatal pathway [5]. Antipsychotic medications block D2 receptors within the basal ganglia, particularly in the striatum, where dopamine normally modulates motor control [5]. Under physiologic conditions, dopaminergic neurons arising from the substantia nigra exert inhibitory control over striatal cholinergic interneurons, maintaining a critical balance between dopaminergic and cholinergic activity [6]. When the D2 receptors are antagonized, this inhibitory dopaminergic influence diminishes, resulting in a relative cholinergic overactivity [6]. This dopaminergic-cholinergic imbalance leads to excessive excitatory output from the basal ganglia to motor pathways, producing sustained, involuntary muscle contractions characteristic of acute dystonia. High-potency antipsychotics are more frequently implicated due to their strong D2 receptor affinity and minimal intrinsic anticholinergic effects [6].

While dystonic reactions commonly involve the cervical, facial, or ocular muscles, involvement of the laryngeal musculature, known as ALD, is a rare but potentially life-threatening condition due to the risk of airway obstruction [7]. Although younger individuals are more frequently affected, ALD has been reported across all age groups, particularly in acute care or hospital settings where rapid drug administration occurs [7]. Risk factors include male gender, younger age, high-potency antipsychotics, parenteral administration, abrupt withdrawal of chronic antipsychotics, and absence of anticholinergic prophylaxis [8].

Clinically, ALD presents with sudden-onset inspiratory stridor, dysphonia, respiratory distress, and occasionally cyanosis, typically within minutes to hours of exposure to the offending agent [3]. Involuntary contraction of the vocal cord and laryngeal musculature leads to functional airway narrowing rather than structural obstruction. Because of its dramatic presentation, ALD can mimic other emergent conditions such as anaphylaxis, laryngospasm, angioedema, asthma exacerbation, post-extubation airway edema, or seizure activity. Failure to promptly recognize ALD may result in unnecessary invasive interventions, including endotracheal intubation, which carry additional morbidity [7]. 

Immediate discontinuation of the offending agent is essential. First-line management consists of parenteral anticholinergic agents such as diphenhydramine or benztropine, which restore dopaminergic-cholinergic balance by reducing acetylcholine-mediated excitatory activity in the striatum. Symptom resolution typically occurs within minutes of administration. Continued oral anticholinergic therapy for 48 hours to one week is recommended to prevent recurrence [8]. In our patient, early recognition and prompt administration of anticholinergic therapy led to rapid reversal of airway compromise and successfully avoided endotracheal intubation. 

Although ALD is infrequently reported in the literature, published cases emphasize its potential severity and the risk of misdiagnosis. Our case adds to existing evidence by demonstrating acute onset airway compromise in an adult patient without prior dystonic history, underscoring that ALD can occur outside the classic high-risk demographic. This case highlights the importance of clinician awareness of this rare but critical adverse effect, and reinforces the need for rapid recognition and treatment to prevent potentially fatal airway obstruction. 

## Conclusions

ALD is an uncommon but potentially life‑threatening extrapyramidal adverse effect of antipsychotic medications, particularly high‑potency dopamine antagonists such as haloperidol. Because its clinical presentation can closely mimic other acute airway emergencies, early recognition is vital. Prompt diagnosis and administration of parenteral anticholinergic medication, such as diphenhydramine or benztropine, can rapidly reverse symptoms and avert airway compromise. Continuing anticholinergic therapy for several days may reduce the risk of recurrence.

This case reinforces the importance of maintaining a high index of suspicion for ALD in patients presenting with sudden stridor or respiratory distress following antipsychotic use. Awareness of this rare condition among intensivists, anesthesiologists, psychiatrists, and emergency physicians can prevent unnecessary and invasive interventions, including endotracheal intubation, thereby markedly reducing morbidity and potential mortality.
